# Effectiveness of Telehealth in Obstetric and Gynecologic Care: A Systematic Review of Health Outcomes

**DOI:** 10.7759/cureus.73144

**Published:** 2024-11-06

**Authors:** Nihal Eltayeb Abdalla Elsheikh, Selma Mohammed Abdelgadir Elhabeeb, Hanady ME M Osman, Ali Hadi M Alhajri, Salem Mesfer S Alsaqoor, Norah Aboud M Alwadai

**Affiliations:** 1 Obstetrics and Gynecology, Najran Armed Forces Hospital, Najran, SAU; 2 Quality and Patient Safety, Najran Armed Forces Hospital, Najran, SAU; 3 Internal Medicine, Najran Armed Forces Hospital, Najran, SAU; 4 Radiology, Najran Armed Forces Hospital, Najran, SAU

**Keywords:** gynecology, health outcomes, obstetric, telehealth, telehealth interventions

## Abstract

Telehealth applications are growing in the fields of obstetrics and gynecology. Strong scientific analysis and clinical recommendations are necessary for these innovative advancements. The purpose of this study was to conduct a thorough evaluation of how telehealth interventions can enhance obstetric and gynecologic health outcomes. Using pre-established inclusion and exclusion criteria, we searched the literature using the Preferred Reporting Items for Systematic Reviews and Meta-Analyses (PRISMA) guidelines. 968 pertinent research articles were found in these databases, and Endnote software checked them for duplication. Only fifty-two of these articles were deemed relevant after a thorough text examination. Every study that was included had its risk of bias evaluated using the Newcastle-Ottawa Scale (NOS). Overall, telehealth interventions enhanced breastfeeding and smoking cessation obstetric outcomes. While preserving mother and fetal outcomes, telehealth interventions reduced the requirement for high-risk obstetric monitoring office visits. According to one study, women with gestational hypertension had lower rates of preeclampsia diagnoses. For continued use of injectable and oral contraceptives, telehealth treatments proved successful; one text-based study discovered higher rates of oral contraception after six months. When medication abortion services were provided via telehealth, access to early abortion was enhanced, and clinical outcomes were comparable to those of in-person care. Few studies have indicated the value of telehealth in improving the notification of test results for sexually transmitted infections and in improving the symptoms of urine incontinence with app-based interventions. Early availability of medical abortion services, breastfeeding, perinatal smoking cessation, telehealth therapies, and high-risk obstetrics schedule optimization were all linked to better obstetric outcomes. More carefully planned research is required to look at these and other interventions in order to produce data that can guide choices regarding the integration of more recent telehealth technology into obstetrics and gynecology practices.

## Introduction and background

The development and deployment of telehealth initiatives are progressing rapidly [1]. Any service related to health that uses electronic communication technologies to provide health or medical information to a patient in a distant or mobile setting is known as telehealth [2].

Telehealth has become a new dynamic for delivering healthcare services, especially with the new global healthcare challenges such as COVID-19 [3]. Specifically, its integration with different branches of medicine with obstetrics and gynecology (OB/GYN) as one of the key areas has provided increased access to care, enhanced patient interaction, and proposed solutions for the issues with location [4]. The use of telehealth services to include virtual consultation, monitoring, and patient education increased showing improved accessibility of healthcare givers to pregnant women, women undergoing gynecological treatment as well as women with Reproductive health issues [5]. Thus, answering questions regarding telehealth as an intervention to support the delivery of these services will help to establish its possible contribution to enhancing the health outcomes of women receiving OB/GYN care [6].

In the context of obstetrics, telehealth has allowed virtual and home-based evaluation of expectant and postpartum women and follow-up of high-risk pregnancies. The transition to virtual care has thus removed many of the typical disadvantages influencing care-seeking, especially for women in remote or shortage areas [7]. Still, concerns have been raised on whether the use of telehealth affects maternal health, for example, the incidences of maternal complications, premature births, and fetal wellbeing. More about the efficiencies of virtual appointments, home-based monitoring tools, and telecommunication platforms in eliminating direct visits, but still providing quality services need to be discussed [8].

Likewise, gynecologic care has noted an increased utilization of telehealth for consultation on diverse conditions; such as menstrual disorders, infertility, menopause, and cancers of the reproductive organs [9,10]. Telehealth is more convenient and flexible for both patient and clinician needs and wants but sufficient evidence to know if telehealth can be as effective as in-face-to-face care is still to be defined [11]. Further, even though telehealth has the potential to enable early diagnosis, effective management of chronic gynecological conditions, and timely management, its feasibility and sustainability need to be studied in detail to assess whether the online service delivery system will supplement face-to-face consultations [12].

Many researchers have investigated the use of telehealth in OB/GYN; however, the results of prior studies are isolated and contradictory. A few provide evidence of positive changes in patient outcomes and patient satisfaction in particular while others depict some issues like technology adoption and usage, issues of telemedicine technology access, as well as the quality of care that can be delivered through remote means [13,14]. Given the current dependence on telehealth, in this field, it becomes necessary to utilize available data and make specific systematic and structured recommendations on the health outcomes related to its application in OB/GYN services [15].

The purpose systematic review is to assess obtained health outcomes to inform about the efficiency of telehealth in OB/GYN and compare outcomes obtained in different studies. This review will be oriented by the examined telehealth impacts on key clinical outcomes, patient satisfaction, and possible concerns to outline its position in contemporary OB/GYN and its perspective on the future of women’s health.

## Review

Methodology

This systematic review of available studies on our topic was conducted according to the PRISMA guidelines (“Preferred Reporting Items for Systemic Reviews and Meta-Analyses”) [16].

Search Strategy

To find published studies in English without consideration of the publishing timeframe, we search five separate databases. We also searched these databases to see if there were any ongoing or prior systematic reviews on the topic. Endnote software (Clarivate, London, UK) was used to combine results from various databases and remove duplicate results. The databases and search strategies used are presented in Table 1.

**Table 1:** Search string used for different databases.

Sr. No.	Database	Search string
1	Scopus	(Telehealth OR Telemedicine OR "Remote Consultation" OR "Virtual Care") AND (Obstetric* OR Gynecology* OR "Women's Health" OR Pregnancy) AND ("Health Outcomes" OR "Clinical Outcomes" OR Efficacy OR Effectiveness)
2	Web of Science	(Telehealth OR Telemedicine OR "Remote Consultation" OR "Virtual Care") AND (Obstetric* OR Gynecolog* OR "Women's Health" OR Pregnancy) AND ("Health Outcomes" OR "Clinical Outcomes" OR Efficacy OR Effectiveness)
3	PubMed/EMBASE	(Telehealth[MeSH Terms] OR Telemedicine[MeSH Terms] OR "Remote Consultation" OR "Virtual Care") AND (Obstetric* OR Gynecolog* OR "Women's Health" OR Pregnancy) AND ("Health Outcomes" OR "Clinical Outcomes" OR Efficacy OR Effectiveness)
4	Google Scholar	(Telehealth OR Telemedicine OR "Remote Consultation" OR "Virtual Care") AND (Obstetric* OR Gynecolog* OR "Women's Health" OR Pregnancy) AND ("Health Outcomes" OR "Clinical Outcomes" OR Efficacy OR Effectiveness)
5	Cochrane Library	(Telehealth OR Telemedicine OR "Remote Consultation" OR "Virtual Care") AND (Obstetric* OR Gynecolog* OR "Women's Health" OR Pregnancy) AND ("Health Outcomes" OR "Clinical Outcomes" OR Efficacy OR Effectiveness)

Studies Selection

During articles extractions, duplicates were removed and every article was extracted and saved in its own Endnote library (Endnote, 2015, Clarivate, London, UK). Two separate reviewers chose which studies to include. While reviewer 2 (HMMO) approved papers based on the data and resolved any arguments on any included research, reviewer 1 (SMSA) reviewed abstracts and titles twice, independently. After a thorough evaluation by reviewers, the publications were selected for inclusion based on the inclusion and exclusion criteria to determine if they provided the pertinent data for the systematic review (Table 2).

**Table 2:** Inclusion and exclusion criteria used for this systematic review.

Questions elements	Inclusion criteria	Exclusion criteria
Study design	Randomized controlled trials, cohort studies, case-control studies.	Opinion pieces, letters to the editor, and conference abstracts without sufficient data.
Population	Studies involving obstetric (pregnancy, prenatal, perinatal, postpartum care) and gynecologic (reproductive health, gynecologic conditions, women’s health) patient populations.	Studies that focus on telehealth interventions for populations outside obstetrics and gynecology (e.g., general medicine, pediatrics).
Intervention	Studies examining telehealth or telemedicine interventions, including remote consultation, virtual care, and digital health technologies used in obstetrics and gynecology.	Studies that do not involve telehealth or telemedicine as the primary intervention.
Outcomes	Studies reporting health outcomes, such as clinical effectiveness, patient satisfaction, healthcare access, treatment adherence, or quality of life.	Studies that do not assess health outcomes related to telehealth (e.g., studies focusing solely on technical aspects without reporting patient outcomes).
Study language	Studies published in English.	Studies published in any other language.

A Microsoft® Excel (Microsoft Corp., Redmond, WA., USA) spreadsheet was used to extract and store data and records.

Risk Bias Assessment

To assess the risk bias of the included studies, the Newcastle-Ottawa Scale (NOS) was employed. Low, moderate, and high assessments were assigned to studies based on selection process bias, intervention bias, departure from intervention bias, missing data bias, outcome bias, and results bias. The inclusion and exclusion criteria were used to calculate preference for selection. Performance bias was assessed through the description of a control arm and the consideration of allocation concealment. Different rankings were given to data management, biased reporting, selective reporting, and full industrial sponsorship. During several sessions, reviewers looked into eligibility limitations and reporting consistency. A second reviewer chose research by taking into account any discrepancies in the reviewers' scores.

Results

Search Results

**Figure 1:**
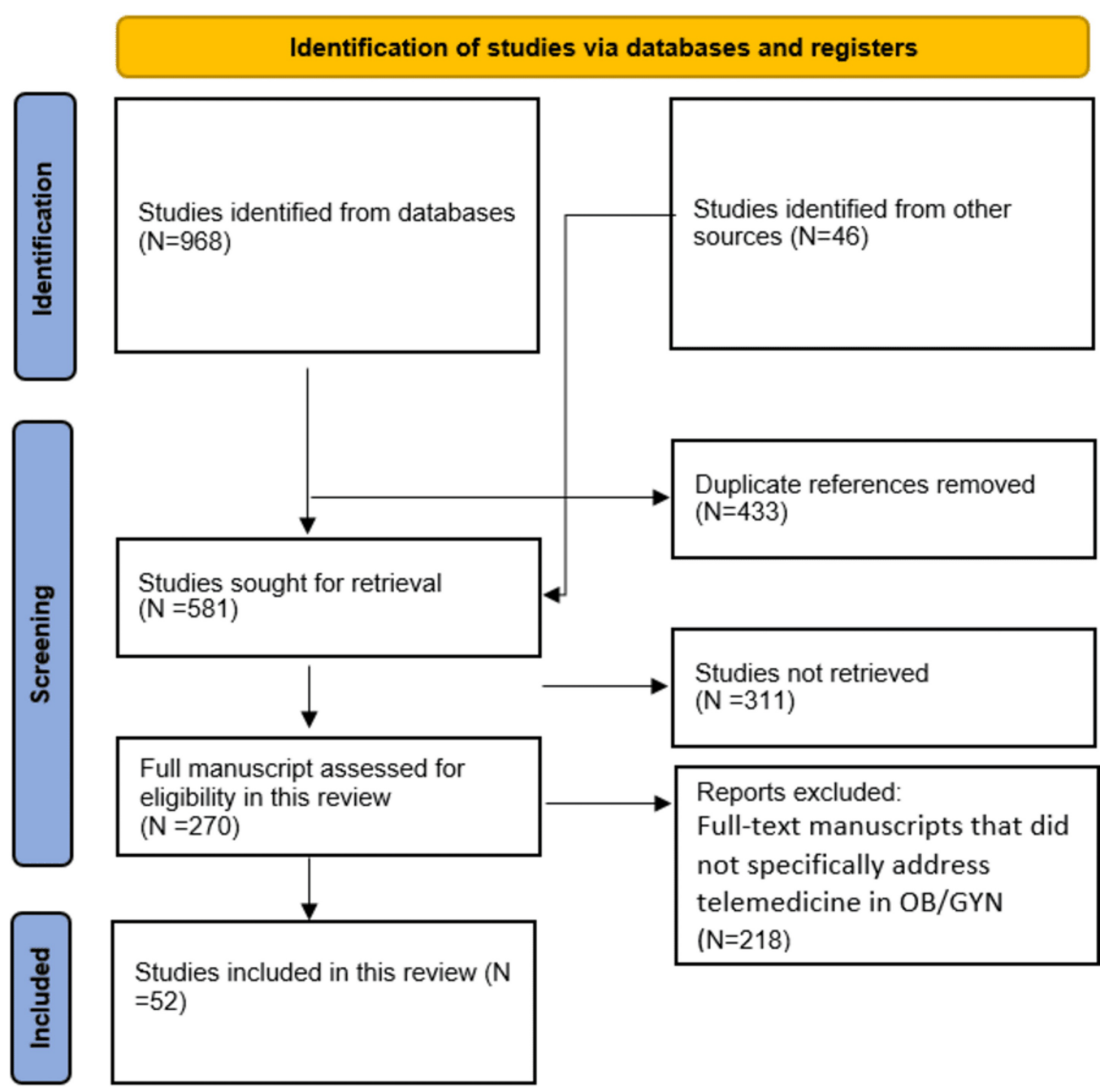
PRISMA flowchart PRISMA: Preferred Reporting Items for Systematic Reviews and Meta-Analyses

Following the studies selection criteria, we identified 1014 studies of which 433 were removed as a duplicate record. We identified 581 studies after the removal of duplicates and 581 were sought for retrieval. Among the studies, 311 out of 581 were excluded as the studies were not retrieved. A total of 270 full-text manuscripts were assessed for eligibility of which 218 were excluded because these studies were not found to specifically address telemedicine in OB/GYN. A total of 52 manuscripts were found to be included in this systematic review (Figure 1).

Risk Bias Assessment

A risk of bias assessment was done using the Newcastle-Ottawa Scale (NOS). Out of 52 studies, 24 studies were found to have low-risk bias, 27 had moderate risk bias and 1 study had high-risk bias. In some studies, a portion of their methodological flaw is the way they chose their controls. Furthermore, no study disclosed the blinding of controls and patients concerning exposure, which may have led to measurement bias (Table 3).

**Table 3:** Risk of bias assessment in the studies included in the systematic review using the Newcastle-Ottawa Scale for case-control studies. Rating scale: 7 to 9 stars = low risk of bias; 4 to 6 stars = moderate risk of bias; 0 to 3 stars = high risk of bias. Selection: (1) If the definition is adequate? (2) If the case representativeness is ok? (3) Controls selection (community or hospital). (4) Controls definitions. Comparability: (1) Comparability of controls and cases according to the analysis or design. Exposure: (1) Exposure determination. (2) The same method for calculation controls and cases. (3) Non-response rate. A single star (★) can be awarded to a study for each numbered item in the exhibit and selection categories. For comparability, no more than two stars (★★) can be given.

Study	Selection				Comparability	Exposure		
	1.	2.	3.	4.	1.	1.	2.	3.
Abroms et al., [17]	★	★			★★	★		★
Tsoh et al., [18]	★	★			★	★	★	
Evans et al., [19]	★	★				★	★	★
Naughton et al., [20]	★	★	★		★★	★	★	★
Yudin et al., [21]	★	★			★★	★	★	★
Stockwell et al. [22]	★	★			★	★	★	★
Moniz et al., [23]	★	★			★★		★	★
Graham et al., [24]	★	★		★	★★	★	★	★
Herring et al., [25]	★	★	★		★		★	★
Dodd et al., [26]	★	★			★★	★	★	★
Huberty et al., [27]	★	★					★	
Fjeldsoe et al., [28]	★	★				★	★	★
Gilmore et al., [29]	★	★	★		★★	★	★	★
Phelan et al., [30]	★	★	★		★	★	★	
Herring et al., [31]	★	★				★	★	
Redman et al., [32]	★	★			★★	★	★	★
Choi et al., [33]	★	★			★	★	★	★
van der Pligt et al., [34]	★	★		★		★	★	★
Jiang et al., [35]	★	★	★		★★	★	★	★
Ahmed et al., [36]	★	★			★★	★	★	★
Gallegos et al., [37]	★	★		★	★	★	★	★
Pérez-Ferre et al., [38]	★	★				★	★	★
Ładyżyński and Wójcicki [39]	★	★	★			★	★	★
Homko et al., [40]	★	★	★			★	★	★
Homko et al., [41]	★	★				★	★	★
Di Biase et al., [42]	★	★			★★	★	★	★
Miremberg et al., [43]	★	★			★	★	★	★
Mackillop et al., [44]	★	★		★			★	★
Dalfrà et al., [45]	★	★	★		★★		★	★
Caballero-Ruiz et al., [46]	★	★			★★		★	★
Lanssens et al., [47]	★	★		★	★		★	★
Zairina et al., [48]	★	★			★★	★	★	★
Hirshberg et al., [49]	★	★	★		★★	★	★	★
Chernick et al., [50]	★	★	★		★★	★	★	★
Tsur et al., [51]	★	★			★		★	
Bull et al., [52]	★	★			★	★	★	★
de Bocanegra et al., [53]	★	★			★★		★	★
Castaño et al., [54]	★	★		★	★★	★	★	★
Trent et al., [55]	★	★	★		★★	★	★	
Hou et al., [56]	★	★			★★	★		
Wilkinson et al., [57]	★	★		★	★	★	★	
Grossman et al., [58]	★	★			★★	★	★	
Grossman et al., [59]	★	★	★		★		★	★
Grossman et al., [60]	★	★			★★	★	★	★
Bracken et al., [61]	★	★	★		★★	★	★	★
Reed et al., [62]	★	★			★★	★	★	★
Huang et al., [63]	★	★	★		★★	★	★	★
Asklund et al., [64]	★	★	★		★	★	★	★
Hoffman et al., [65]	★	★	★		★★	★		★
Haggerty et al., [66]	★	★			★★	★	★	★
Joseph et al., [67]	★	★	★		★	★	★	★
(Vonk Noordegraaf et al., [68]	★	★			★★	★	★	★

GRADEpro GDT (Grading of Recommendations Assessment, Development, and Evaluation pro Guideline Development Tool) indicated that the studies that were part of this meta-analysis had low quality of evidence. The inclusion of observational studies (case-control), which increases the risk of bias because it is unable to randomize the exposure, and the inconsistent nature of the research were the main causes of the low quality of the evidence.

Characteristics of Included Studies

This systematic review covers almost all categories of telehealth interventions in obstetrics and gynecology with emphasis on RCTs originating from the USA, UK, Australia, and other countries. All but two of the studies evaluated the impact of telehealth delivery modes including SMS and mobile applications, and telemedicine in improving maternal health.

All but one study used an RCT approach to assess the impact of interventions on selected health outcomes such as prenatal health, obesity prevention, vaccination, length of breastfeeding, gestational diabetes, use of contraception, and maternal mental health. The studies included low-investigator trials of less than 100 subjects and multisite evaluations of patients with different disorders.

Most of the research was performed in the USA, whereas others were from Australia, the UK, Israel, Spain, Poland, and China which covered a vast geographical location. This distribution demonstrates the worldwide interest in the use of telehealth to meet maternal and neonatal healthcare needs for developed and developing care systems (Table 4).

**Table 4:** Characteristics of included studies. RCT: randomized controlled trials, GWG: gestational weight gain, HEI: Healthy Eating Index, PA: physical activity, SMS: Short Message Service, WIC: women, infants, and children, IOM: institute of medicine, GDM: gestational diabetes mellitus, RM: remote monitoring, GHD: gestational hypertension disorder, SUI: stress urinary incontinence, PFMT: pelvic floor muscle training.

Author	Year	Study design	Country	Key findings
Abroms et al., [17]	2017	RCT	USA	Findings show that the Quit4baby SMS messaging program is effective in the initial stages and towards the end of pregnancy, yet not in the following delivery phase.
Tsoh et al., [18]	2010	RCT	USA	At both prenatal visits throughout the intervention period, participants were more inclined to get tobacco use guidance from their providers (60.9 versus. 15.8%, p = 0.003).
Evans et al., [19]	2014	RCT	USA	A good program that provides lessons for upcoming mHealth initiatives is Text4baby. This extensive study showed how the program's messaging to women during the research period affected their attitudes and beliefs in the beginning.
Naughton et al., [20]	2017	RCT	UK	When combined with regular NHS cessation therapy, a text-messaging service may help pregnant smokers quit.
Yudin et al., [21]	2017	RCT	USA	The majority of women who received text messages expressed satisfaction; only 15 out of 129 (12%) said they did not enjoy receiving them, and 24 out of 129 (19%) said the material was not useful.
Stockwell et al., [22]	2014	RCT	USA	Text messaging was linked to higher influenza vaccination rates with this low-income obstetric group, particularly among those who received texts early in the third trimester.
Moniz et al., [23]	2013	RCT	USA	Overall, 32% of participants had received an influenza vaccination; there was no difference between those in the Overall (31% (n=31)) and Flu (33% (n=34)) categories (difference 1.7%, 95% CI −11.1 to 14.5%).
Graham et al., [24]	2017	RCT	USA	An original empirical contribution to conducting evaluations for independent Internet-based treatments is the characterization of the usage patterns of the control and intervention arms. GWG results were linked to specific usage patterns in the intervention arm.
Herring et al., [25]	2017	RCT	USA	Results indicate that among economically deprived African American women who are obese, a combined prenatal and postpartum weight management intervention improves 6-month weight outcomes.
Dodd et al., [26]	2018	RCT	Australia	The average difference between the HEI scores at 28 and 36 weeks during pregnancy was 0.01 (confidence interval (CI (−2.29, 2.62)) and −1.16 (CI (−4.60, 2.28)), respectively. The availability of the app for smartphones did not significantly increase the HEI score (p =.452) in any way.
Huberty et al., [27]	2017	RCT	USA	Regardless of time or frequency, physical activity did not rise. More inactive time and more activity reductions were observed in those who received 6 PA SMS per week. SMS might not be a "potent" enough tactic to boost PA.
Fjeldsoe et al., [28]	2010	RCT	Australia	In post-natal women, exposure to the intervention led to an increase in the frequency of PA or walking for exercise.
Gilmore et al., [29]	2017	RCT	USA	When compared to standard care provided by the current WIC program, the E-Moms intervention did not, on average, reduce postpartum weight retention among women receiving benefits. Nonetheless, there exists some evidence that suggests better weight management would result from increased intervention adherence.
Phelan et al., [30]	2017	RCT	USA	Weight change during a 12-month period, as determined by baseline, 6-month, and 12-month measurements, was the main outcome. Changes in nutrition and physical activity, as well as the percentage of people who returned to their preconception weight, were secondary outcomes.
Herring et al., [31]	2016	RCT	USA	When compared to standard care, the intervention decreased the percentage of women who went above and beyond IOM guidelines (37% vs. 66%, P = 0.033). Pregnancy-related weight increase was lower for intervention participants (8.7 vs. 12.3 kg, corrected mean variation: −3.1 kg, 95% CI: −6.2 to −0.1). There were no group variations in the obstetric or neonatal outcomes.
Redman et al., [32]	2017	RCT	USA	Mobile phones can be used to administer an intensive lifestyle change for GWG in a way that is both economical and scalable.
Choi et al., [33]	2016	RCT	USA	At the 12-week mark, the group receiving the intervention reported feeling less of a barrier to being active, such as a lack of energy (p = 0.02) than the control group. During the 12-week trial period, fewer people used the mobile app to use the daily journal and respond to daily messages.
van der Pligt et al., [34]	2018	Case-control	Australia	The study's online intervention appears to have the potential to lower postpartum women's waist circumference.
Jiang et al., [35]	2014	RCT	China	The length of exclusive breastfeeding was the main outcome. The length of exclusive breastfeeding in both the interventions and the controls was compared using survival analysis.
Ahmed et al., [36]	2016	RCT	USA	A web-based collaborative breastfeeding monitoring system could be an effective strategy for increasing breastfeeding length, exclusivity, and intensity.
Gallegos et al., [37]	2014	Retrospective cohort study	Australia	The duration of exclusive breastfeeding seems to be improved by completely automated text messaging services. The program offers a well-received, individualized support service that gives women the ability to take charge of their breastfeeding problems.
Pérez-Ferre et al., [38]	2010	RCT	Spain	The method produces comparable results for pregnancy, birth, and the newborn while drastically lowering the requirement for outpatient clinic visits.
Ładyżyński and Wójcicki [39]	2007	RCT	Poland	Despite the significantly greater (15 times) reported frequency in the at-home telecare group, the mean level of metabolic regulation and the insulin dose modification patterns were quite similar in both groups. Due to the considerable workload associated with daily data analysis and the excessive within-day variability in glycaemic control, the patient-collected data were not effectively utilized.
Homko et al., [40]	2007	RCT	USA	Due to their occasional use of the telehealth system, the benefit of Internet-based blood glucose monitoring for impoverished women with GDM was limited. Women in the telehealth group felt more psychosocially capable of managing their diabetes, even though system use was not linked to better pregnancy outcomes.
Homko et al., [41]	2012	RCT	USA	Maternal blood glucose levels and newborn weight did not significantly differ between the two groups (telehealth vs. controls).
Di Biase et al., [42]	1997	RCT	Italy	Higher insulin dosages in the DIANET group compared to the conventional treatment were linked to the outcomes, and both groups had a marked decrease in hypoglycemia reactions. According to the results, telemedicine-DIANET is a useful method of offering specialized treatment during pregnancy.
Miremberg et al., [43]	2018	RCT	Israel	The implementation of a smartphone-based weekly communication and feedback platform between the interdisciplinary diabetes-in-pregnancy clinic team and patients with gestational diabetes mellitus enhanced glycemic control and patient compliance while reducing the need for insulin administration.
Mackillop et al., [44]	2018	RCT	UK	It is safe for women with GDM to have their blood glucose levels monitored remotely. We used GDm-health to show out our improved data collecting capabilities. Women favored this treatment paradigm even though glucose control and neonatal and maternal results were comparable.
Dalfrà et al., [45]	2009	Non-randomized control experiment	Italy	The number of examinations at the clinics for diabetes was lower for both telemedicine groups. Women with gestating diabetes had better pregnancy outcomes when using a telemedicine platform for glucose monitoring, and all diabetic pregnancies had better quality of life.
Caballero-Ruiz et al., [46]	2017	RCT	Spain	Clinicians spent 27.389% less time evaluating patients, and they saw 88.556% fewer in-person visits per patient. Patients expressed great satisfaction with the system, believing it to be well-managed and beneficial.
Lanssens et al., [47]	2017	Retrospective cohort	Belgium	One promising approach in prenatal care is an RM follow-up for women with GHD. It creates the possibility of reversing the existing trend of prenatal interventions, which would result in more interventions and, consequently, more medicalized prenatal care.
Zairina et al., [48]	2016	RCT	Australia	Pregnancy-related asthma quality of life and asthma control may be enhanced by telehealth programs that assist self-management.
Hirshberg et al., [49]	2018	RCT	USA	Compared to typical office-based follow-up, text-based surveillance is more effective in acquiring blood pressure readings and achieving current clinical criteria in the immediate post-discharge interval for women with pregnancy-related hypertension.
Chernick et al., [50]	2017	RCT	USA	Adolescent girls in the emergency room found a pregnancy preventive texting strategy to be both feasible and acceptable.
Tsur et al., [51]	2008	RCT	Israel	There were 108 women enrolled, 50 in the treatment group and 58 in the control group. It is not common for women to utilize contraceptives as advised. Just two women in every group used two forms of contraception, and after three months, 50% of the study population and 40% of the group serving as the control were using contraception (p = 0.41).
Bull et al., [52]	2016	Cluster RCT	USA	The costs of the intervention were an extra $126 per person (+10.6%) on top of the $1184 expenses of the control program. For the entire sample, there were zero statistically important variations in the primary outcomes. Compared to the control group (6.72%; P =.02), Hispanic individuals in the intervention condition reported fewer pregnancies at monitoring (1.79%).
de Bocanegra et al., [53]	2017	Prospective cohort study	USA	Reminders had a good effect on users of contraceptive injections, but generally, the Bedsider reminders had no effect on timely return for injections and refills.
Castaño et al., [54]	2012	RCT	USA	Compared to routine care alone, the introduction of daily instructional text messages improved OCP continuation at six months. To enhance continuation in one, ten women would require this straightforward intervention.
Trent et al., [55]	2015	RCT	USA	In terms of increasing clinic attendance for visits involving relatively long-acting reversible contraception, the DepoText intervention is viable, acceptable, and has demonstrated short-term preliminary efficacy.
Hou et al., [56]	2010	RCT	USA	Oral contraceptive adherence was not enhanced by daily text message reminders. Objectively, the rate of missing pills was remained quite high in both groups, even though the lack of effect might have been caused by the control group's frequent use of alternate reminder systems.
Wilkinson et al., [57]	2017	RCT	USA	After sexual activity, emergency contraception is an effective and secure way of preventing pregnancy, however its effectiveness wanes with time. When EC is taken before the need arises, it can be taken shortly after unprotected sexual activity, which maximizes its effectiveness.
Grossman et al., [58]	2011	Cohort study	USA	According to 25% of telemedicine patients, they would have liked to see the doctor in person. Preferring face-to-face contact was substantially correlated with nulliparity, younger age, and lower levels of education. The frequency of problems reported by telemedicine patients during the investigation period did not change significantly.
Grossman et al., [59]	2013	Cohort study	USA	Telemedicine has the potential to decrease second-trimester abortions and increase access to medical abortion, particularly for women in distant places.
Grossman et al., [60]	2017	Cohort study	USA	Medical abortion seldom results in adverse events, and when it comes to clinically relevant adverse events, telemedicine is not worse than in-person care.
Bracken et al., [61]	2014	RCT	USA	It is possible and, for the majority of women, preferable to a clinic visit to follow up by remote communication following a medical abortion.
Reed et al., [62]	2014	RCT	USA	In a pediatric emergency room, STI notification levels among female adolescents—but not male adolescents—were enhanced by obtaining a private phone number and utilizing call + text messaging.
Huang et al., [63]	2015	RCT	USA	While women who participated in this randomized trial who were given device-guided tranquil respiration reported some improvement in the frequency and intensity of their hot flashes, the paced metabolism intervention was much less successful compared to a music-listening interference in reducing these symptoms.
Asklund et al., [64]	2017	RCT	Sweden	Women with SUI responded well to the mobile app treatment, which produced improvements that were clinically significant. Adherence to PFMT and access to initial therapy may both be improved by this app.
Hoffman et al., [65]	2017	RCT	Sweden	With the use of the Tät® app, self-management of stress urine incontinence produced notable and clinically meaningful long-term results and may be used as a first-line treatment.
Haggerty et al., [66]	2016	RCT	USA	It is possible to use technology to help women suffering Type I cancer of the endometrium or hyperplasia lose weight. Weight loss resulted from both therapies, although the effects of more interpersonal engagement were more pronounced. Weight loss was linked to decreases in IL-2 expression.
Joseph et al., [67]	2015	RCT	USA	Reduced sedentary behavior, increased physical activity at light to moderate lifestyle intensities, improved psychosocial outcomes, and elevated participant satisfaction were all linked to a culturally relevant physical activity program that was delivered via Facebook and text messages.
Vonk Noordegraaf et al., [68]	2014	RCT	Netherlands	Following gynecological surgery, women who use the eHealth intervention return to work more quickly, have better quality of life, and experience less discomfort.

The studies as presented showcased a significantly better or worse performance of telehealth in enhancing health status by the different telehealth interventions. Other mobile interventions like Quit4baby and Text4Baby properly intervened in the health-related behaviors during pregnancy and influenced smoking cessation and vaccination respectively. In this aspect, they seem to have produced mixed results regarding their effects on postpartum results. Mobile interventions for weight loss and diabetes had certain efficacies when applied in certain population subsets including; those who were extremely low-income earners or those with gestational diabetes. However, findings were relative to the general increase in physical activity and improvements in diet. Reviews on medical abortion tele-support and contraceptive use suggested that remote monitoring of the clients was quite acceptable. Specifically, telemonitoring of blood glucose in gestational diabetes and telemanagement of contraception brought increased access and satisfaction from the patients. Interventions that included components of automated messaging and web-based platforms for support were effective in the trial in lengthening the time to exclusive breastfeeding and enhanced postpartum weight loss results. In addition, the interventions relating to breastfeeding knowledgeable mothering revealed having positive effects on the proportion of breast milk feeding and maternal satisfaction.

These studies offer integrated reviews and knowledge advancement about the effectiveness of telehealth interventions in OB/GYN to enhance the health of women. Nevertheless, the study established that telehealth was beneficial in all the above areas despite low results on postpartum care from the intervention, specifically the SMS-based programs. This result suggests the necessity of further integrated work to support health gains that go beyond the prenatal period.

Discussion

The fact that our study screened almost 968 published articles and that the final review contained 52 articles with 31,967 participants was significant in the developing field of telehealth. To reflect the entire range of telehealth in women's health services, the review covered both low- and high-risk obstetrics, reproductive health, and general gynecology. We identified some interesting telehealth therapies that merit clinical adoption and further research, despite the paucity of solid evidence in favor of these interventions in OB/GYN.

One pattern that came through our systematic review is that text messaging could be useful for reinforcing the use of contraception, breastfeeding, and quitting smoking during pregnancy, among other health behaviors. However, it proved ineffective to use text messaging to start a new therapy or behavior, like switching to a different method of contraception. The success of telehealth programs like text messaging may depend critically on patient motivation, and further study is required to pinpoint populations that might be more responsive to behavior-change interventions [6].

The use of virtual visits and remote monitoring in situations where facility-based treatment is impeded is another issue addressed by our study. We discovered that in the instance of high-risk obstetrics, fewer planned outpatient visits for the treatment of diabetes and hypertension resulted from patient-generated data sent via mobile phones and remote monitoring. Telehealth made early abortion more accessible in the instance of pharmaceutical abortion. In both cases, the telemedicine service's efficacy and safety were on par with in-person treatment [4,69,70]. Future research should look at how these interventions might be used in other services, like managing and providing contraception, that some patients might find challenging to access.

Additional studies are required to examine these themes, paying particular emphasis to matching the telehealth modality with the health outcome that stands to gain the most from a focused intervention, a few of which are included here. The most recent facets of telehealth, such as wearable technology and remote monitoring, which have so far mostly been investigated through pilot or feasibility studies, require more RCTs. Furthermore, this systematic review did not look at cost analysis, provider ratings, or patient happiness, all of which can help guide telehealth's future developments. Our research has several advantages. First, only RCTs, comparative cohort studies, and case-control studies with a minimum of two comparison groups were included. Second, the findings apply to practicing women's health care professionals due to the emphasis on clinical and behavioral outcomes. Lastly, the systematic review approach provides useful insights from the expanding and varied body of research on telehealth therapies being investigated in gynecology and obstetrics. This review offers a preliminary framework that can be modified in light of new information. The need for more research to determine which therapies are successful and ought to be used in clinical practice is highlighted by the possible advantages and evidence gaps.

One of the review's limitations is that we limited our search method to peer-reviewed studies and did not include gray literature, which limited the amount of material that could be found and limited access to adverse trials. Evidence from RCTs, comparative observational studies, and case-control studies had to be compiled in the review. Because observational studies, including those that employed self-reporting procedures that might have introduced bias, pose design-specific risks to internal validity, the evidence from these research should be evaluated with greater caution. The evidence synthesis's scope was restricted to English-only studies carried out in nations with extremely high UN Human Development Index scores. This review is therefore less applicable to poorer countries. Women who agreed to take part in tiny, targeted studies or randomized trials may also not be broadly applicable to the broader public. We were also unable to ascertain if certain effects might be temporary or the consequence of Hawthorne effects due to the varying research follow-up dates. Our development procedure differed from the PRISMA methodology, as was indicated in the Methods section. Due to the extensive extent of our evaluation, we did not publish our review process before the start of the study since we evaluated the caliber and substance of the literature before establishing inclusion and exclusion criteria.

However, we included information about our methodology that improves the study's transparency and reproducibility, and every step of the review was controlled to guarantee uniformity between topic groups throughout. Our synthesis offers a thorough and thorough examination of the impacts of telehealth throughout several domains of female reproductive health care by concentrating on the RCT evidence and restricting our review to investigate designs that compared women subjected to and unaffected by telehealth interventions.

This review demonstrates the significant knowledge gap about telehealth-mediated interventions in women's health care, with a few noteworthy exceptions. Little is known about the potential advantages or disadvantages of these interventions, even though the emergence of technology advancements has made it possible to incorporate telehealth into practice. To help physicians decide how to incorporate telehealth into their medical practice in manners that enhance patient care, more data is required. Further, well-designed studies are required to examine interventions like wearable technology and virtual visits to encompass the broader adoption of telehealth in obstetrics and gynecology, although this review indicates some benefits for particular telehealth interventions, particularly text messaging and remote monitoring.

## Conclusions

The findings of this systematic review can be of significant value and support the potential use of telehealth in improving the delivery of obstetric and gynecological care through output-based technology including remote monitoring and text messaging. From 52 studies surveyed, totaling over 31,000 participants, our included study indicated telehealth potential approaches including text messaging to support healthy behaviors, drinking, as well as breastfeeding and contraception without desire for sex, and monitoring obstetric high-risk situations. However, potential areas are still lacking sufficient data, primarily on behavior initiation using telehealth and the overall incorporation of newer technologies, including wearable gadgets.
